# Improved method for efficient imaging of intracellular Cl^−^ with Cl-Sensor using conventional fluorescence setup

**DOI:** 10.3389/fnmol.2013.00007

**Published:** 2013-04-10

**Authors:** Perrine Friedel, Piotr Bregestovski, Igor Medina

**Affiliations:** ^1^Inserm Unité 901Marseille, France; ^2^Aix-Marseille UniversitéMarseille, France; ^3^INMEDMarseille, France; ^4^Inserm UMR 1106, Brain Dynamics InstituteMarseille, France

**Keywords:** fluorescent biosensors, intracellular chloride, non-invasive monitoring, Cl-Sensor, KCC2, neuron, patch clamp

## Abstract

Chloride (Cl^−^) homeostasis is known to be fundamental for central nervous system functioning. Alterations in intracellular Cl^−^ concentration ([Cl^−^]_i_) and changes in the efficacy of Cl^−^ extrusion are involved in numerous neurological disorders. Therefore, there is a strong need for studies of the dynamics of [Cl^−^]_i_ in different cell types under physiological conditions and during pathology. Several previous works reported having successfully achieved recording of [Cl^−^]_i_ using genetically encoded Cl-Sensor that is composed of the cyan fluorescent protein (CFP) and Cl^−^-sensitive mutant of the yellow fluorescent protein (YFP_Cl_). However, all reported works were performed using specially designed setups with ultra-sensitive CCD cameras. Our multiple attempts to monitor Cl^−^-dependent fluorescence of Cl-Sensor using conventional epifluorescence microscopes did not yield successful results. In the present work, we have analysed the reason of our failures and found that they were caused by a strong inactivation of the YFP_Cl_ component of Cl-Sensor during excitation of the CFP with 430 nm light. Based on the obtained results, we reduced 20-fold the intensity of the 430 nm excitation and modified the recording protocol that allows now stable long-lasting ratiometric measurements of Cl-Sensor fluorescence in different cell types including cultured hippocampal neurons and their tiny dendrites and spines. Simultaneous imaging and patch clamp recording revealed that in mature neurons, the novel protocol allows detection of as little as 2 mM changes of [Cl^−^]_i_ from the resting level of 5–10 mM. We demonstrate also a usefulness of the developed [Cl^−^]_i_ measurement procedure for large scale screening of the activity of exogenously expressed potassium-chloride co-transporter KCC2, a major neuronal Cl^−^ extruder that is implicated in numerous neurological disorders and is a target for novel therapeutical treatments.

## Introduction

A rapidly growing amount of evidence shows the importance of neuronal chloride (Cl^−^) homeostasis for the functioning of central nervous system. The changes in the ability of neurons to normally regulate the intracellular Cl^−^ concentration ([Cl^−^]_i_) are implicated in several neurological disorders including brain recovery from acute trauma and stroke, different types of epilepsies and neuropathic pain. In most of the mentioned pathologies, changes in neuronal chloride homeostasis are related to modification in the activity of the Cl^−^ extruder KCC2 (potassium-chloride co-transporter II) and Cl^−^ importer NKCC1 (sodium-potassium-chloride co-transporter I) (Kahle et al., 2008; Blaesse et al., 2009). Both co-transporters belong to the family of twelve transmembrane domain electroneutral cation-chloride co-transporters (CCCs). The electroneutrality of CCCs does not allow direct measurement of their activity. To circumvent this problem, researchers estimate the functionality of CCCs by analysing the consequences of their ion transport activity. While in heterologous expression systems many laboratories used ^86^Rb fluxes as read-out of the CCC activity (Payne, 1997), in neuronal cells the activity of CCCs was determined so far by analysing the changes in Cl^−^ homeostasis (reviewed by Medina and Chudotvorova, 2006; Blaesse et al., 2009). Historically, most pioneer works in the field estimated the level of [Cl^−^]_i_ by analysis of the reversal potential of Cl^−^-permeable GABA or glycine receptor-coupled channels (GABAR, GlyR) (Rivera et al., 1999; Cohen et al., 2002; Khirug et al., 2008; Tyzio et al., 2008). The recent achievements in cell biology and microscopy promoted the use of different Cl-sensitive fluorescent probes for analysis of neuronal Cl^−^ homeostasis (Bregestovski et al., 2009; Berglund et al., 2011). The first set of markers were Cl-sensitive dyes (Quinolinium Cl^−^ indicators) using capability of halides to quench the fluorescence of heterocyclic organic compounds with quaternary nitrogen (Chen et al., 1988; Verkman, 1990). These compounds have relatively good sensitivity and selectivity to Cl^−^. They have been used for measurements of [Cl^−^]_i_ in a variety of preparations (see review Bregestovski et al., 2009), however, they are prone to strong bleaching and present a significant leakage rate (Inglefield and Schwartz-Bloom, 1997; Nakamura et al., 1997).

A more recent and promising method for non-invasive analysis of Cl^−^ is based on the halide-binding properties of yellow fluorescent protein (YFP) and its derivatives (Wachter and Remington, 1999; Jayaraman et al., 2000). These Cl-sensitive biosensors have a number of advantages: (1) they show much more stable fluorescence at long-lasting monitoring; (2) they allow excitation in the visible range of wavelength; (3) they can be targeted to specific cell types (4) and they have a high molecular weight, which prevents the diffusion of the indicators from cells. The weak point of YFP-based molecules is their sensitivity to intracellular pH (pH_i_) and some organic anions (Jayaraman et al., 2000) that should be taken into account during analysis of YFP-emitted fluorescence.

The first ratiometric Cl^−^ indicator was designed by fusion of YFP with cyan fluorescent protein (CFP) through a polypeptide linker (Kuner and Augustine, 2000). In this construct, called Clomeleon, CFP acts as a reference point for normalizing expression levels. Later was developed a more sensitive probe, called Cl-Sensor, composed of CFP and mutated form of YFP with higher Cl^−^ sensitivity (YFP_Cl_, the apparent *EC*_50_ ~ 30–50 mM) (Markova et al., 2008; Waseem et al., 2010). Using this probe, we have successfully characterized changes in [Cl^−^]_i_ in different cell lines (Markova et al., 2008; Waseem et al., 2010) and in cultured hippocampal and spinal cord neurons (Waseem et al., 2010; Pellegrino et al., 2011). However, in these studies, the measurements of [Cl^−^]_i_ were performed on a specially designed fluorescence setup, for convenience called thereafter ultrasensitive setup that was equipped with a highly sensitive CCD camera and a low output intensity excitatory unit (polychromatic light selector equipped with attenuation filters). Our attempts to monitor [Cl^−^]_i_ from the same samples of cultured cells using a conventional epifluorescence setup described in the present study gave non-satisfactory results due to continuous changes in the fluorescence emitted by YFP_Cl_ under resting conditions and, therefore, to strong drifts of the ratio baseline.

In the present work, we studied the dependence of the photostability of Cl-Sensor on different excitation wavelengths and discovered a previously undescribed process of transient inactivation of the YFP_Cl_ component of Cl-Sensor with illumination by short wavelength blue light (430 nm), commonly used to excite the reference CFP component of the Cl-Sensor. Based on the obtained results, we modified the setup and recording protocol to allow long-lasting ratiometric recording of the fluorescence from different cell types expressing Cl-Sensor.

## Materials and methods

All manipulations with animals were performed in agreement with the guidelines of the Animal Care and Use Committee of INSERM (Institut National de la Santé et de la Recherche Médicale).

### Cell lines and transfection

The majority of the experiments were performed using mouse neuroblastoma cells (N2a) (ATCC, #CCL-131). The cells were cultured in Dulbecco's modified Eagle's medium supplemented with 10% fetal bovine serum, 100 units/ml penicillin and 100 μg/ml streptomycin. For experiments, we used exclusively culture passages from 3 to 10.

For Cl-Sensor fluorescence recording, N2a cells were transfected with a mixture of at least two different pcDNAs encoding: (1) Cl-Sensor in gw1 vector (Waseem et al., 2010) and (2) human α1 subunit of glycine receptor (GlyR) (Waseem et al., 2010). The activation of Cl^−^ permeable GlyR under depolarized conditions allowed the induction of the rise of [Cl^−^]_i_. To study the extrusion capacity of KCC2, N2a cells were transfected with a mixture of three pcDNAs: (1) Cl-Sensor, (2) GlyR, (3) cherry-KCC2. For control experiments, cherry-KCC2 was substituted with empty vector pcDNA3.1 (mock transfected cells). The cherry-KCC2 construct was made on the basis of previously described eGFP-KCC2 (Pellegrino et al., 2011) with cherry replacing eGFP.

For transfecting cells growing on coverslips in 35 mm dishes, 300 μl of Opti-MEM media were mixed with 7 μl of Lipofectamine reagent 2000 (Life Technologies) and 1.5 μg of different pcDNAs pre-mixed in desired proportions. The mixture was incubated for 30 min at room temperature (RT) and distributed thereafter above cells. After 2 h of incubation at 37°C, half of the medium was replaced with fresh medium. Cells were used for the experiments 2–3 days after transfection. The described proportions of pcDNA/Lipofectamine and incubation times allowed obtaining a moderate expression of the exogenous proteins in approximately 60–80% of cells. We avoided increasing the efficacy of transfection to 100% in order to prevent overproduction of the protein and preserve healthiness of the cells.

The proportions of pcDNAs for transfection were as follows: 0.3 μg Cl-Sensor + 0.3 μg cherry-KCC2 (or pcDNA3.1) + 0.6 μg GlyR. Such co-transfection resulted in the expression of Cl-Sensor and GlyR in 100% of cherry-KCC2 positive cells.

### Primary cultures of rat hippocampal neurons

Hippocampal neurons from 19 days Wistar rat embryos were dissociated using trypsin and plated onto poly-ethylenimine-coated coverslips at a density of 70,000 cells cm^−2^ in minimal essential medium (MEM) supplemented with 10% NU serum (BD Biosciences, Le Pont de Claix, France), 0.45% glucose, 1 mM sodium pyruvate, 2 mM glutamine, and 10 IU ml^−1^ penicillin–streptomycin as previously described (Buerli et al., 2007). On day 9 of culture incubation, half of the medium was changed to MEM with 2% B27 supplement (Life Technologies).

The neurons used in the present study were transfected with only Cl-Sensor. The increase in [Cl^−^]_i_ was achieved using the activation of the endogenous GABA_A_ receptors with isoguvacine under depolarizing conditions. Transfections of 10 day *in vitro* (DIV) neuronal cultures were performed as described previously (Buerli et al., 2007). For transfection of cultures in 35 mm dishes, 300 μ l of Opti-MEM media were mixed with 7 μl of Lipofectamine reagent 2000 (Life Technologies), 1 μl of Magnetofection CombiMag (OZ Biosciences, France) and 1.5 μg of Cl-Sensor pcDNAs. The mixture was incubated 20 min at RT and thereafter distributed dropwise above the neuronal culture. Culture dishes were placed on a magnetic plate (OZ Biosciences) and incubated 40 min at 37°C. Transfection was terminated by the substitution of 70% of the incubation solution with fresh culture media. Neurons were used in experiments 3 days after transfection.

### Imaging setup

**Figure 1:**
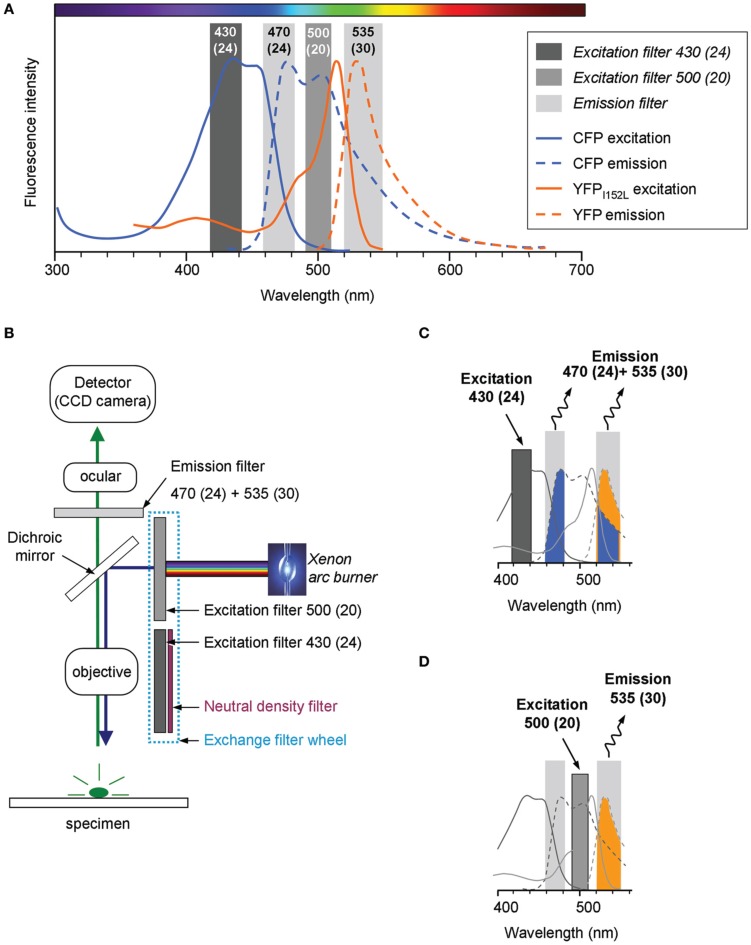
**Light pass of conventional epifluorescence setup. (A)** Excitation and emission spectra of the filter set used in the study (vertical bars) overlapped with emission and excitation spectra for cyan and yellow fluorescent proteins carrying the I152L mutation (CFP and YFP_I152L_, respectively). The plot is a schematic presentation of spectral bandpasses of the setup designed based on the information provided by the filter set manufacture (Chroma Technology Corp., USA). The absorbance spectra of YFP_I152L_ is reproduced from Galietta et al. (2001). The absorbance spectra of CFP and emission spectra of CFP and YFP were taken from R. Tsien's website (http://www.tsienlab.ucsd.edu/Documents.htm). **(B)** Scheme of the setup. The light source (Xenon arc burner) was connected to a filter wheel including two excitation filters and one neutral density filter (ND filter) placed in front of 430(24) nm filter. The emitted fluorescence was recorded using a digital CCD camera. **(C)** Schematic presentation of the recorded portion of fluorescence emitted by Cl-Sensor during excitation with a 430(24) nm filter (see Materials and Methods for details). **(D)** Schematic presentation of the fluorescence signal emitted by Cl-Sensor during excitation with a 500(20) nm filter.

The Cl-Sensor is a chimera protein composed of the yellow fluorescent protein with enhanced sensitivity to Cl^−^ (YFP_Cl_) that is linked to the CFP (Markova et al., 2008). The emission and excitation spectra of CFP and YFP_Cl_ strongly overlap (Figure 1A). To obtain the effective separation of the fluorescence emitted by CFP and YFP_Cl_, we used the ECFP/EYFP single band exciters filter set (#59217, Chroma Technology Corp., Bellows Falls, VT, USA). The excitation filters from the set [430(24) nm and 500(20) nm] were mounted into the Lambda 10-B Filter wheel (Sutter Instruments Company, Novato, USA). Notice that the first number indicates median wavelength and the number in parentheses indicates the wideness of the bandpass. In front of 430(24) nm filter was mounted a 5.0% transmission neutral density filter (ND 1.3 B–5% Trans, Chroma Technology Corp.). The emitted fluorescence was collected through a unique double bandpass filter 470(24) + 535(30) nm using an inverted Olympus fluorescence microscope (IX71, Olympus, France). The fluorescence was excited using an X-Cite Series 120Q light source (Lumen Dynamics Group Inc., Ontario, Canada). Image recording was performed using a CoolSNAP*HQ* Monochrome CCD camera and Metamorph software that includes a multi-dimensional acquisition option (MDA) (Roper scientific sas, Evry, France) (see Figure 1B for scheme of the lightpass).

During the excitation of the Cl-Sensor with 430(24) nm filter the emitted fluorescence signal passing through 470(24) + 535(30) nm filter was composed of a CFP component (colored in blue in the scheme shown in Figure 1C) and an YFP_Cl_ component (colored in yellow). In addition, the signal emitted at 535(30) nm might also contain the fluorescence of the YFP component excited by Fluorescence Energy Resonance Transfer (FRET) from CFP. The illumination of Cl-Sensor, using a 500(20) nm filter, induced excitation of only YFP_Cl_ component that was recorded through the 535(30) band of the emission filter (colored in yellow) (Figure 1D).

### Fluorescence recording procedure

Coverslips with transfected cells were placed onto the inverted microscope and perfused with an external solution (in mM): 140 NaCl, 2.5 KCl, 20 Hepes, 20 D-glucose, 2.0 CaCl_2_, 2.0 MgCl_2_, pH 7.4. To induce cell depolarization and increase [Cl^−^]_i_, we replaced NaCl with an equimolar amount of KCl (the final concentration of KCl is mentioned in results and figure legends for each type of experiments). All solutions included 10 μM bumetanide, a selective blocker of sodium-potassium-chloride co-transporter NKCC1, to prevent chloride influx through this transporter. When needed, glycine, an agonist of GlyR (final [C] = 50 μM) or isoguvacine, an agonist of GABA_A_ receptors (final [C] = 30 μM) were added to the solution with increased concentrations of KCl. The times of application are mentioned in results and figure legends. The osmolarity of the final solutions was carefully verified and adjusted to 310 mOsm using NaCl or KCl.

Unless otherwise specified, the frequency of acquisition was 0.05 Hz and the duration of excitation was 20–50 ms during most of the experiments. The increase in [Cl^−^]_i_ during the experiment was achieved using a fast perfusion system described previously (Medina et al., 1994) allowing rapid change of the solution (within 100 ms) in the entire optical field. The inner diameter of the quartz perfusion tubes was 250 μm.

All recordings from N2a cells were performed using a LUCPlanFLN 20× Objective, NA 0.45 (Olympus, France) that allowed simultaneous recordings from 30 to 50 transfected cells. Recordings from neurons were performed using either described above 20× objective for the monitoring of fluorescence from soma and proximal dendrites or 40× objective [LUCPlanFLN, NA 0.60 (Olympus, France)] used for visualization of tiny dendrites and spines.

All experiments were performed at 24–25°C.

### Simultaneous imaging and electrophysiology recording

For simultaneous imaging of Cl-Sensor and patch-clamp recording, neurons were bathed in the described above external solution containing in addition 0.5 μM of TTX (Na^+^ channels blocker) that was applied to prevent spontaneous neuronal activity. The recording micropipettes (5 MΩ) were filled with a solution containing (in mM): 150 KCl, 10 HEPES, 20 mg/ml gramicidin A (dissolved in DMSO), pH 7.2. Isoguvacine (30 μM) was dissolved in external solution and focally applied to soma and proximal dendrites through a micropipette (same as for patch clamp) connected to a Picospritzer (General Valve Corporation, pressure 5 psi). The resting membrane potential (E_m_) was determined by switch to the “I = 0” mode. Recordings were made using an Axopatch-200A amplifier and pCLAMP acquisition software (Axon Instruments). Data were low-pass filtered at 2 kHz and acquired at 10 kHz. Input resistance (*R*_in_) and capacitance were routinely determined from the analysis of responses to hyperpolarizing/depolarizing steps of −10/+10 mV applied from the holding potential of −80 mV; in this range, the *I–V* curve of cells was close to linear. Values of the membrane potential were corrected for series resistance during analysis.

The image acquisition started 1–2 min after formation of the gigaseal and continued without any interruption through the whole experiment. The synchronization of the image and patch clamp recordings was made manually by insertion of the tags at desired moments of the recordings.

#### Statistical analysis

All results are shown as mean ± SEM. The statistical significance was calculated using Anova or Kolmogorov–Smirnov tests as indicated.

## Results

To characterise the properties of Cl-Sensor, we used routinely mouse neuroblastoma N2a cells transfected with mixture of pcDNAs encoding Cl-Sensor and GlyR. The GlyR, a receptor-gated Cl^−^ channel, was employed to modify rapidly and selectively [Cl^−^]_i_ and, as consequence, produce changes in Cl-Sensor responses. We performed also some initial experiments using Chinese hamster ovary (CHO-K1) and PC12 (rat pheochromocytoma) cell lines that showed similar responses to activation of GlyR. However, the experiments involving exogenous expression of the neuronal potassium-chloride co-transporter KCC2 showed higher efficacy of the KCC2 expression in N2a, than in PC12 or CHO-K1 cells. Therefore, N2a cells were chosen as model for the study of Cl^−^ dynamics using Cl-Sensor.

### Use-dependent inactivation of Cl-Sensor

**Figure 2:**
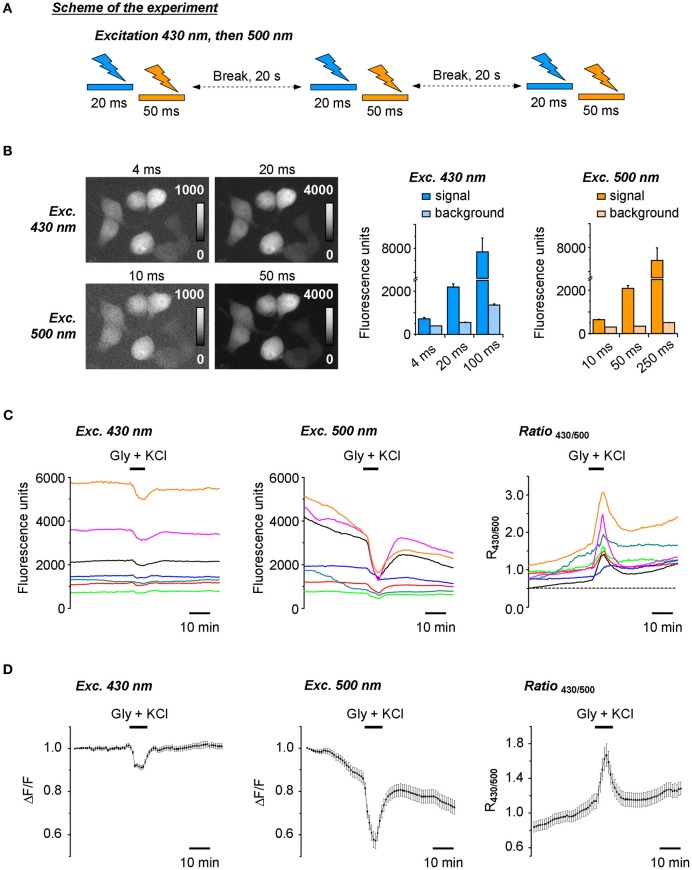
**Ratiometric recording of the Cl-Sensor fluorescence using a non-adjusted fluorescence setup. (A)** Scheme of the protocol of experiment. Cl-Sensor was excited every 20 s using two consecutive light pulses through first 430 nm and then 500 nm excitation filters. **(B)** Examples of fluorescence images obtained by excitation at 430 nm and 500 nm at different exposure times as indicated. Plots illustrate the mean ± SEM of fluorescence intensity measured on the transfected cells (signal) and region free of cells (background) (*n* = 15 regions measured in the same sample). **(C)** Fluorescence responses of individual cells to 5 min application of the external solution containing 50 μM glycine and 100 mM KCl. Examples were chosen to illustrate the variability of fluorescence intensity and signal ratio. Ratio values (R_430/500_) were obtained by arithmetic division of fluorescence induced by Cl-Sensor excitation via 430 and 500 nm filters. **(D)** Left and middle plots show normalized mean ± SEM fluorescence responses from 36 cells recorded in the optical field during excitation with 430 nm (left) and 500 nm (middle) filters, respectively. Values were obtained as difference of each individual point with first recorded value of the experiment (Δ F) divided per first recorded value (F). The right plot illustrates mean ± SEM of signal ratio R_430/500_. The first points on the plots correspond to the first image taken in the experiment.

At the initial stage of the work, a ratiometric recording of Cl-Sensor fluorescence was made using the setup described in Figure 1 and materials and methods, but without ND filter introduced into 430(24) nm light path. The excitation of cultured cells expressing Cl-Sensor was performed every 20 s through first 430(24) and then 500(20) nm narrow-band excitation filters (referred thereafter as 430 nm and 500 nm filters for convenience) (Figure 2A). The acquisition parameters for the fluorescence responses, induced by excitation at 430 nm (F_430_), were selected as such, providing in average a 10:1 signal noise ratio and good spatial resolution of the transfected cells (Figure 2B, upper right image and left plot). The lowering of the acquisition time to 4 ms resulted in strong loss of the resolution of transfected cells due to the decrease of the signal to noise ratio. The acquisition parameters for the fluorescence excited at 500 nm (F_500_) were adjusted to obtain a F_500_ intensity similar to that of F_430_ (Figure 2B, lower right image and right plot). In the experiments described in Figures 2B–D, the selected acquisition times were 20 ms and 50 ms for F_430_ and F_500_, respectively, and the CCD camera binning was 2 × 2.

We constantly observed that using the described above protocol, the F_430_ was stable in most of the recorded cells during at least 30 min, while the F_500_ continuously decreased starting from the beginning of the recording (Figures 2C,D, left and middle plots). As a consequence, the ratio of F_430_/F_500_ responses (R_430/500_) progressively increased (Figures 2C,D, right plots). The artificial increase in [Cl^−^]_i_, achieved by the co-application of KCl and glycine, produced a further increase of R_430/500_ that only partially decreased after washout of the applied drugs. Due to a continuous increase of R_430/500_ at resting conditions and the variability of this change in different experiments, we were unable to perform a comparative ratiometric analysis of the Cl-Sensor emitted fluorescence under different experimental conditions.

The observed drift of the basal level of YFP fluorescence and related change of fluorescence ratio were not due to changes in [Cl^−^]_i_ as similar cells analysed using the previously described setup using a short time of excitation (Markova et al., 2008; Waseem et al., 2010) did not show a drift of the baseline (data not shown, but see Figure 5 for a stable recording from similar cells using a modified conventional setup). We suggested, therefore that the progressive change of the YFP_Cl_ fluorescence is a result of use-dependent photoinactivation of the YFP_Cl_ component of Cl-Sensor.

**Figure 3:**
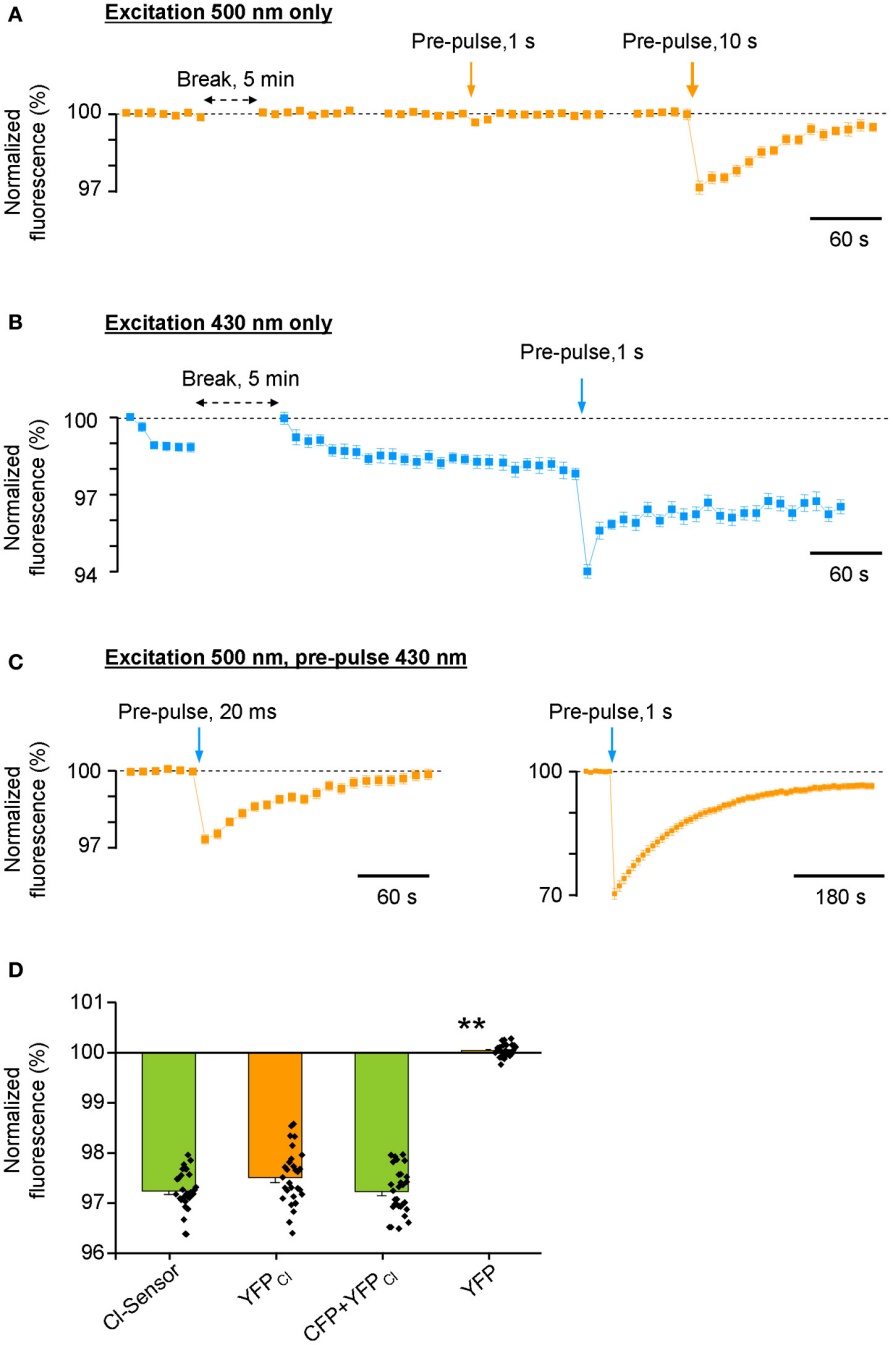
**Differential photoinactivation of CFP and YFP components of Cl-Sensor.** All responses shown in the figure are mean ± SEM values from 40 to 43 cells visualized in a single optical field. Similar data were observed in 8 other experiments. In all depictured experiments, the duration of test excitation was 20 and 50 ms through 430 or 500 nm filters, respectively. The interval between acquisitions was 10 s (unless otherwise indicated with horizontal double head arrow). **(A)** Fluorescence responses of Cl-Sensor to repetitive excitations through a 500 nm filter. Vertical arrows show moments of Cl-Sensor excitation with additional light pulses through the same filter. **(B)** Repetitive excitation of Cl-Sensor through a 430 nm filter induces rapid decline of the fluorescence. Vertical arrow indicates additional excitation using the same 430 nm filter. **(C)** Repetitive excitation of Cl-Sensor through a 500 nm filter. Blue arrows indicate application of additional light pulses through a 430 nm filter. Notice the different ordinate and abscise axes resolution in right plot. **(D)** Comparison of the degree of inactivation of *F*_500_ fluorescence by 20 ms pulse of 430 nm light [similar protocol as shown in **(C)**, left plot] in cells expressing Cl-sensor, YFP_Cl_, 1:1 mixture of CFP + YFP_Cl_ and YFP. Mean ± SEM, 3 experiments, 10 cells per experiment. ^**^p < 0.01.

Surprisingly, we found that the repetitive excitation of the Cl-Sensor only through the 500 nm bandpass filter did not produce a progressive decrease of YFP fluorescence (Figure 3A). A 20-fold longer pulse (1 s) of 500 nm light decreased transiently YFP fluorescence by only 0.34 ± 0.14%. A much longer pulse (10 s) of 500 nm light induced a 3.0 ± 0.25% decrease of YFP fluorescence that progressively recovered to the basal level within 3 min. Thus, the excitation of Cl-Sensor through the 500 nm filter could not be considered as a factor in the drift of the basal line of fluorescence in Figure 2B. On the contrary, repetitive cell stimulation with 430 nm light pulses (20 ms every 10 s) induced a rapid decline of F_430_ to a steady-state level of 97–98% (Figure 3B). The observed decline was 430 nm light dependent: the elongation of the interval between excitation pulses to 5 min allowed full signal recovery while a long illumination of the cells (1 s) caused an additional, partially reversible, decrease of F_430_ intensity (Figure 3B).

Next, we performed excitation of Cl-Sensor using only the 500 nm filter, with a single 20 ms pulse of 430 nm light introduced during recording, to mimic the double-wavelengths excitation protocol used in the previous ratiometric experiments (Figure 3C). This short 430 nm excitation pulse induced a sizable decrease of the F_500_ (2.6 ± 0.18%, 36 cells). A longer pulse (1 s) of 430 nm light induced a strong inhibition of the F_500_ by 30 ± 1.3%. It should be noted that the recovery of fluorescence from inactivation was only partial and lasted several minutes. Overall, in three analysed experiments, the single 20 ms pre-pulse of 430 nm light produced 2,8 ± 0.70% inhibition of F_500_ (Figure 3D, see column “Cl-Sensor”). Thus, even a relatively short illumination of the Cl-Sensor by 430 nm light causes a potent inactivation of its YFP_Cl_ fluorescent component.

We wondered whether the observed F_500_ inactivation was an intrinsic property of YFP_Cl_ or the result of the association of YFP_Cl_ in a molecular complex with CFP. To answer this question, we made three additional transfections of N2a cells with (1) YFP_Cl_ alone; (2) mixture of pcDNAs encoding CFP and YFP_Cl_; (3) original non-mutated form of YFP. We observed that 430 nm light pulses inhibited F_500_ fluorescence emitted by YFP_Cl_ alone to a similar extend than with Cl-Sensor (Figure 3D). Consistently, the cells expressing a mixture of CFP and YFP_Cl_ proteins also showed similar degree of F_500_ fluorescence inhibition. By contrast, the F_500_ fluorescence in cells expressing non-mutated YFP was fully resistant to illumination with 430 nm light. We therefore concluded that 430 nm light-induced inactivation of Cl-Sensor is a result of YFP molecule mutation. A future more detailed study is necessary to characterize the physical process inducing 430 nm-dependent inactivation of YFP_Cl_ and discover which out of the three mutations increasing Cl^−^ sensitivity of YFP_Cl_ (H148Q, I152L, and V163S) are responsible for this process.

**Figure 4:**
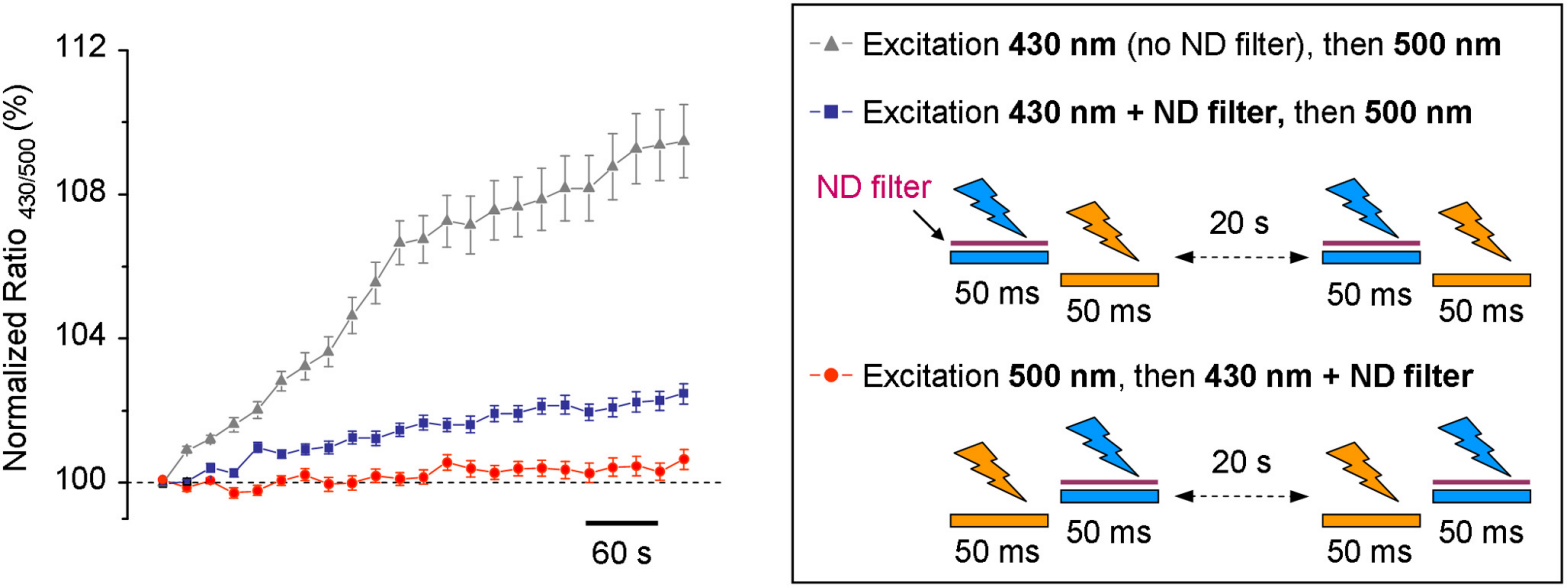
**Time-dependent change of the R_430/500_ at resting conditions measured using different protocols.** The trace with triangles illustrates the results obtained using the non-modified light pass and protocol shown in Figure 2A. The traces with blue squares and red circles were obtained using protocols shown in the right inset. ND filter, neutral density filter.

**Figure 5:**
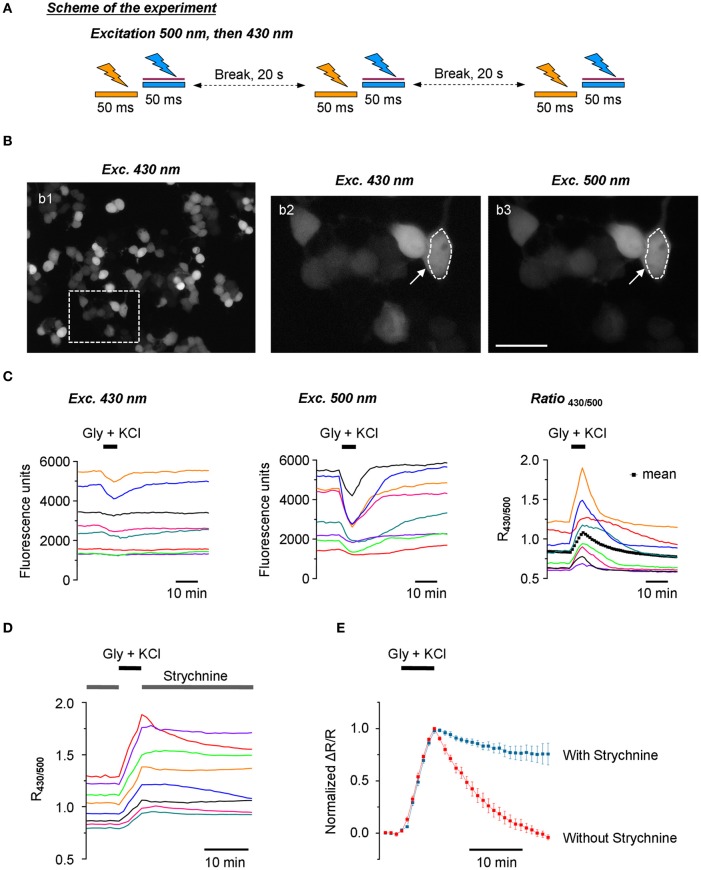
**Stable recording of Cl-Sensor fluorescence using the updated setup. (A)** Scheme of the experiment: Cl-Sensor was excited every 20 s using two consecutive 50 ms light pulses through first a 500 nm and then a 430 nm excitation filter. The sequence of excitation was different as compared to Figure 2A. **(B)** Example of N2a cells transfected with Cl-Sensor and excited at 430 nm (panel b1). CCD camera binning was settled 4 × 4. The image in panel b2 shows a digitally amplified region from b1. Image in b3 shows fluorescence obtained using 500 nm excitation (CCD binning 2 × 2). The arrow indicates an example of a region of interest (ROI) drawn around a cell to measure fluorescence intensity. Scale 20 μm. **(C)** Left and middle plots illustrate recordings of fluorescence induced using 430 nm and 500 nm excitation pulses. The right plot shows corresponding traces of signal ratio (R_430/500_). Each color corresponds to the same cell shown in the three plots. The artificial increase of [Cl^−^]_i_ was achieved by application of an external solution containing glycine (50 μM) and KCl (100 mM). The black dotted line in the right plot illustrates a mean value of 42 cells recorded in the depictured experiment. **(D)** Dynamics of R_430/500_ change in an experiment similar to the one depictured in **(C)** but in the presence of Strychnine, a blocker of glycine receptors (0.3 μM) in the control external solution. Notice the slow or inexistent recovery of the R_430/500_ value after wash out of glycine and KCl. **(E)** Comparison of the kinetics of the recovery of R_430/500_ (mean ± SEM) in the experiments depictured in panels **(C)** (right plot, 42 cells) and **(D)** (38 cells).

Based on our results, we introduced a 1:20 ND filter into the 430 nm light pass (see methods and scheme in Figure 1B). As this filter strongly reduced the excitation light and, by consequence, decreased the intensity of the emitted fluorescence signal, we increased binning factor to 4 × 4 and settled acquisition time of F_430_ to 50 ms. The acquisition parameters of F_500_ remained unchanged. Time lapse recording with modified light pass revealed that the use-dependent R_430/500_ fluorescence ratio drift became much weaker, but did not completely disappear (Figure 4). To further reduce the inhibitory effect of having the 430 nm light pulse preceding F_500_, we changed the order of the excitation wavelengths: during each ratiometric measurement, the cells were illuminated first using 500 nm filter and immediately after using 430 nm filter. Such modification in the sequence of excitation wavelengths elongated the interval between 430 nm and 500 nm excitations from few milliseconds to 20 s (compare protocols depictured on the scheme in Figure 4) and resulted in additional reduction of the use-dependent modulation of R_430/500_ (Figure 4, red circles; see also Figure 5C for long-lasting recording).

### Stable recording of Cl-Sensor fluorescence

Using the protocol with modified sequence of excitation wavelengths and introduced ND filter to 430 nm lightpass (Figure 5A), we performed a study of the dynamic of Cl-Sensor fluorescence in N2a cells expressing exogenous GlyR. To avoid Cl-Sensor inactivation with 430 nm light, the selection of the recording field and focusing on the cell surface were performed using weak transparency white light. The image acquisition parameters were chosen using additional samples of cells and were kept identical through the experiment. For this purpose, we first selected the acquisition parameters for F_500_ allowing the obtaining of a good spatial resolution of the transfected cells and a 10:1 signal-noise ratio in average (Figure 5B, panel b3). For most experiments, the acquisition time was from 50 to 100 ms and CCD camera binning 2 × 2. After obtaining the F_500_ image of the desired quality, a single F_430_ image was taken with an acquisition time of 50 ms and binning 4 × 4 (Figure 5B, panels b1 and b2). The average intensities of fluorescent cells at F_430_ and F_500_ were than compared and times of F_500_ acquisition were adjusted to obtain R_430/500_ ratio close to 1. Under such conditions, it was possible to record stable level of Cl-Sensor fluorescence for several hours at both F_500_ and F_430_ channels (not shown). Both F_430_ and F_500_ fluorescence signals were chloride sensitive: the application of an external solution containing 100 mM KCl and 50 μ M glycine induced a transient decrease of F_430_ and F_500_ (Figure 5C). However, as shown in Figure 5C (left and middle panels), the magnitude of the F_430_ change was more than 3-fold lower than the magnitude of the F_500_ change in the same cells (the mean ± SEM decrease of F_430_ was 6.2 ± 1.0 %, compared to 21.6 ± 2.6% for F_500_. *n* = 3, 15 cells per experiment). The ratio of Cl-Sensor fluorescence signals, R_430/500_, quantified for the same cells, was stable at resting conditions and increased by 21.2 ± 3.7% following induction of Cl^−^ influx (Figure 5C, right panel). In most of the cells, the observed increase of R_430/500_ was transient with a 6.8 ± 0.8 min half recovery time (*n* = 3, 45 cells, data not shown). The application of 1 mM furosemide, a diuretic inhibiting most of the Cl^−^ extruders, did not affect recovery time (not shown). By contrast, the application of 0.3 μM of strychnine, a selective antagonist of GlyR, elongated almost 8-fold fluorescence recovery after the induction of an increase in [Cl^−^]_i_: the estimated half recovery time was 56.6 ± 5.3 min (*n* = 29 cells) (Figures 5D,E). This effect may result from the presence of a small amount of glycine in the physiological solution. Indeed, it has been shown that in physiological solutions prepared using distilled water, the background levels of glycine, determined by HPLC, were 40–50 nM and this type of contamination is extremely difficult to avoid (Lerma et al., 1990). To exclude the possible contribution of GlyR to Cl^−^ extrusion, all further experiments were performed in a control external solution containing 0.3 μM strychnine.

**Figure 6:**
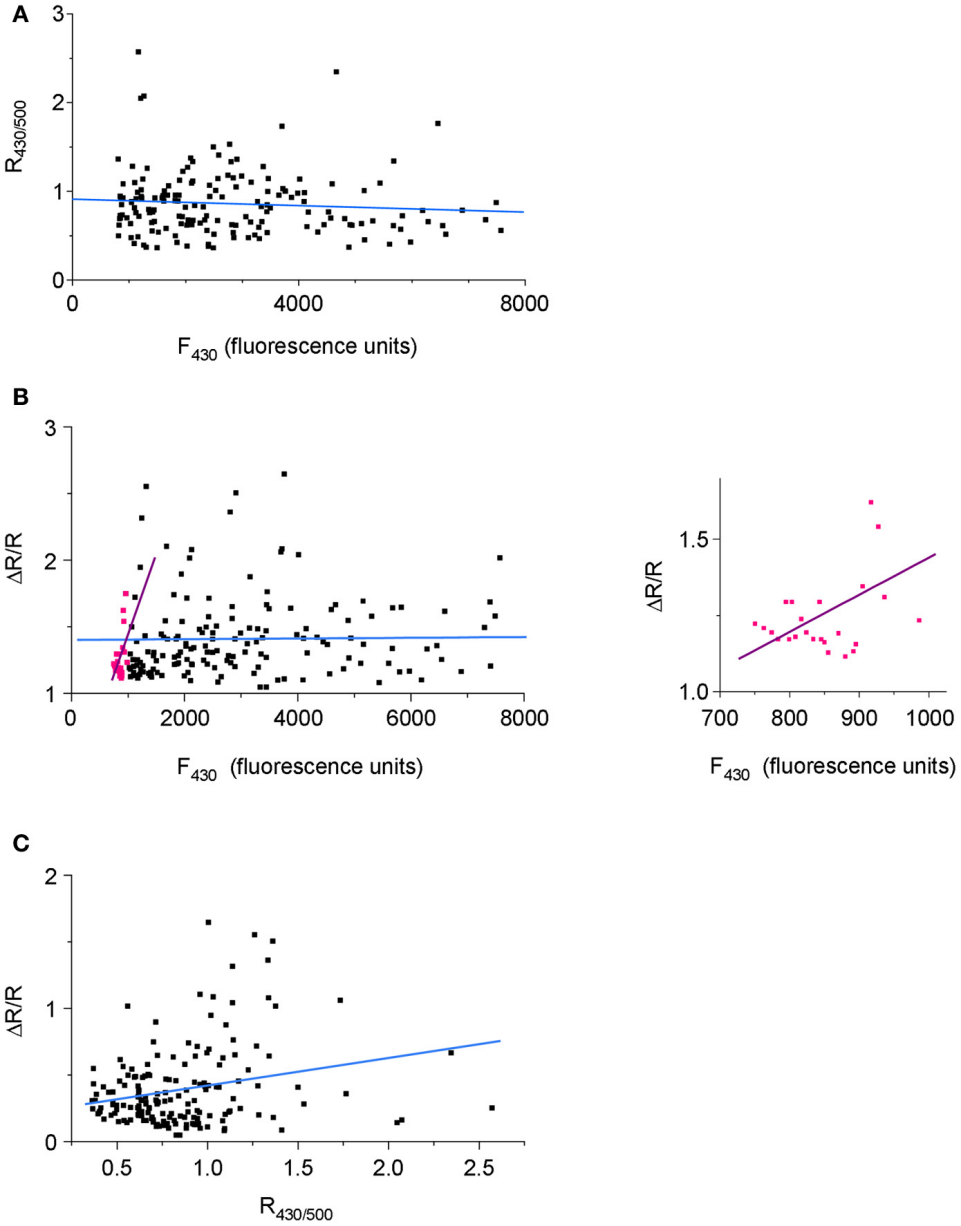
**Dependence of the variability of R_430/500_ on Cl-Sensor protein expression.** Pooled data from four experiments (40–45 cells per experiment). **(A)** A scatterplot of R_430/500_ as function of the intensity of Cl-Sensor fluorescence induced at 430 nm (F_430_). **(B)** Correlation between the magnitude of Cl-Sensor response to imposed increase of [Cl^−^]_i_ (co-application of Glycine and KCl; ΔR/R) and the F_430_. Pink dots illustrate ΔR/R values measured in range of 700–999 relative units of fluorescence. Black dots show ΔR/R in range of 1000–8000 units. The straight lines are best linear fits of the corresponding cell populations. The inset plot shows the distribution of pink points from principal plot at different time base. **(C)** Correlation between the magnitude (Δ R/R) response and basal level of R_430/500_.

One of the characteristic features of Cl-Sensor fluorescence measured in N2a cells [as well as in PC12 and CHO-K1 cells (not shown)] was a high variability of both F_430_ and F_500_ fluorescence intensities, as well as calculated R_430/500_ ratio (Figure 5C). Presumably, the F_430_ fluorescence was proportional to the level of Cl-Sensor protein expression (Cl-resistant CFP component), whereas the F_500_ fluorescence characterized both the level of protein expression and the effect of the intracellular milieu (i.e. [Cl^−^]_i_, pH_i_, organic anions) quenching YFP_Cl_ fluorescence (see Figures 1C,D for spectra and Jayaraman et al. (2000) for sensitivity of YFP to different anions). To verify whether the level R_430/500_ depends on the level of Cl-Sensor protein expression, we performed a Pearson's correlation analysis between the intensity of F_430_ fluorescence and R_430/500_ values. We found the absence of the statistically significant correlation between these two parameters (*r* = −0.103, *p* = 0.08, *n* = 184, Figure 6A). The analysis of the relative change of R_430/500_ in response to imposed [Cl^−^]_i_ rise (ΔR/R) revealed be-modal correlation: in cells expressing low level of Cl-Sensor protein (F_430_ ranging 700–1000 relative fluorescence units) we detected statistically significant positive correlation (*r* = 0.48, *p* = 0.008, *n* = 23, Figure 6B), whereas in cells expressing higher amount of Cl-Sensor protein (F_430_ ranging from 1000 to 8000 relative fluorescence units), there was no statistically significant correlation between ΔR/R and F_430_ (*r* = 0.006, *p* = 0.22 1, *n* = 161, Figure 6B). To take this observation in consideration, all future experiments were performed on cells exhibiting a F_430_ fluorescence in the range from 1000 to 8000 relative fluorescence units.

During analysis of Cl-Sensor responses to applications of glycine under depolarizing conditions, we notices that cells with higher level of R_430/500_ showed higher magnitudes of Δ R/R responses (see individual traces in Figures 5C,D). The correlation analysis confirmed the existence of significant positive correlation between these two parameters (*r* = 0.29, *p* = 0.0001, *n* = 161, Figures 6C). Thus, cells showing higher basal level of R_430/500_ respond better to an increase in [Cl^−^]_i_.

### Recording of KCC2 activity

**Figure 7:**
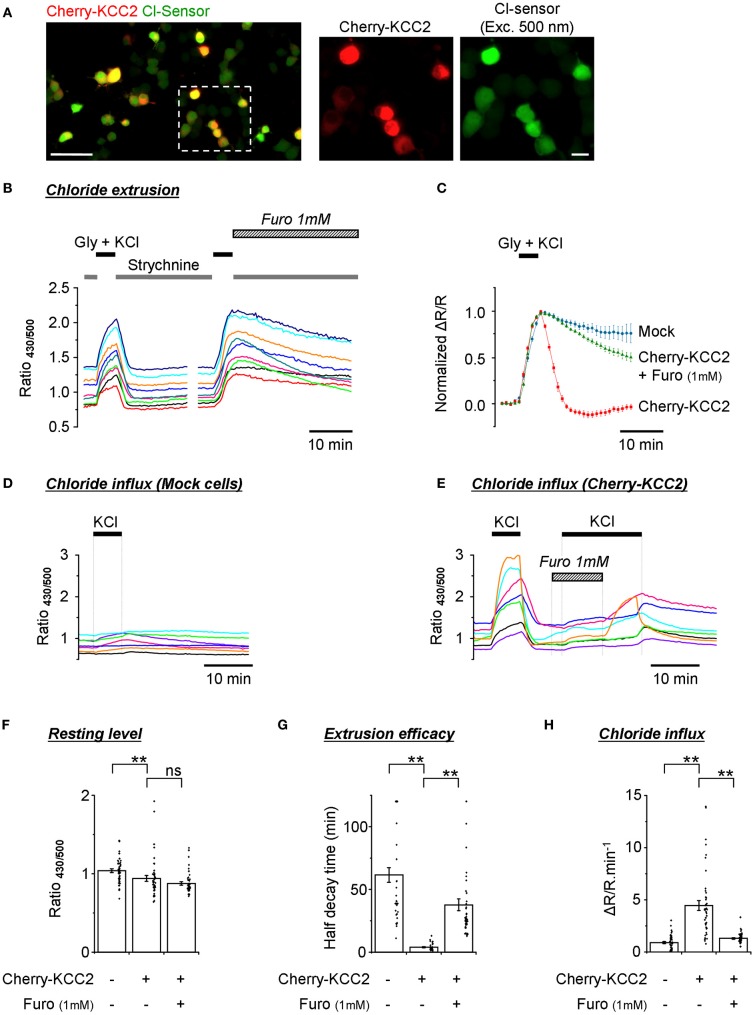
**Monitoring of the activity of exogenously expressed KCC2 using the improved Cl-Sensor method. (A)** The left image illustrates a typical merge image of N2a cells cotransfected with Cl-Sensor (green) and cherry-KCC2 (red). The middle and right images show expression of cherry-KCC2 and Cl-Sensor at higher digital magnification. Scale 50 μm in left panel and 10 μm in middle and right panels. **(B,C)** Analysis of the kinetics of R_430/500_ recovery after an artificial increase of [Cl^−^]_i_ induced by the application of an external solution containing glycine (50 μM) and KCl (100 mM). Plot **(B)** shows examples of R_430/500_ traces recorded from individual cells in control conditions and in the presence of Furosemide, a wide-range cation-chloride transporters blocker, as indicated. Plot **(C)** shows a comparison of the kinetics of R_430/500_ recovery in mock transfected cells and cells expressing cherry KCC2 under control conditions and in the presence of Furosemide (1 mM). **(D,E)** Examples of R_430/500_ recordings in individual mock-transfected cells [Cl-Sensor + empty pcDNA3 vector, panel **(D)**] or cells expressing Cl-Sensor + cherry-KCC2 **(E)** in response to the application of an external solution containing 140 mM of KCl. In this set of experiments, cells did not express GlyR. Note that application of 140 mM of KCl does not affect R_430/500_ in mock-transfected cells but induces a strong increase of R_430/500_ in cells expressing cherry-KCC2. The application of Furosemide prevents the KCl-induced rise of R_430/500_. The wash out of Furosemide in the presence of KCl partially restored an increase of R_430/500_. **(F)** Quantification of the resting R_430/500_ in N2a cells expressing cherry-KCC2 in the presence or absence of Furosemide and mock-transfected cells (experiment similar to those depictured in Panels **D**,**E**). In Furosemide, the measurements were made 5 min after the start of drug application. **(G)** Estimation of the extrusion capacity of the KCC2 transporter. Plot shows quantification of half decay time of R_430/500_ after an artificial increase of chloride in N2a cells as shown in panel **(B)**. **(H)** Estimation of Cl^−^ influx mediated by the KCC2 transporter. Plot shows the magnitude of ratio increase during application of 140 mM KCl as shown in panels **(D)** and **(E)**. Measurements were made 30 s before KCl application and 5 min after the start of KCl application. Bars **(F**–**H)** are mean ± SEM values (*n* = 3, 15 cells per experiment). Black points show individual values. ^**^p < 0.01, Kolmogorov–Smirnov test.

One of the most important applications for Cl^−^ sensitive fluorescent markers is the screening of mammalian expression libraries and the discovery of compounds that modify the activity of CCCs–important targets of therapeutical treatments (Kahle et al., 2008; Blaesse et al., 2009). In previous work performed using an ultrasensitive setup, we have successfully used Cl-Sensor to monitor changes in the activity of the neuronal potassium-chloride co-transporter KCC2 (Pellegrino et al., 2011). In the present study, we verified whether a modified conventional epifluorescence setup could also allow the effective recording of the efficacy of Cl^−^ extrusion by exogenously expressed KCC2. For this purpose, we created a chimera protein composed of cherry fluorescent protein and rat KCC2 (see Materials and Methods for details) and expressed it into the N2a cells together with Cl-Sensor and GlyR. Red fluorescence of cherry-KCC2 allowed the easy identification of cells expressing the chimera protein (Figure 7A).

A 5 min application of the external solution with 100 mM KCl and 50 μM glycine induced a strong increase of R_430/500_ that declined rapidly (within 2 min) to its original resting level in all cells expressing cherry KCC2 (Figure 7B). Moreover, in many cells the R_430/500_ value after recovery was even lower than at the beginning of experiment, thus resulting in the appearance of the characteristic after-recovery transient decrease of R_430/500_ (Figure 7C, red trace). The rapid decrease of R_430/500_ was dependent on the activity of KCC2, as the application of furosemide (1 mM) strongly delayed R_430/500_ recovery after wash-out of KCl and glycine (Figures 7B,C, and G).

A previous study had characterized KCC2 as an electroneutral transporter with its direction and magnitude of net K^+^-Cl^−^ cotransport depending on the sum of K^+^ and Cl^−^ chemical potential differences (Payne, 1997). We therefore made an attempt to record KCC2-mediated Cl^−^ influx by increasing the extracellular concentration of KCl to 140 mM (no GlyR expressed in this set of experiments). Under such conditions, both the Cl^−^ and K^+^ chemical gradients were directed intracellularly. Figure 7D illustrates that, in mock transfected cells, the application of KCl did not modify or slightly increased R_430/500_. The overall increase was 0.9 ± 0.1 relative units (*n* = 3, 30–45 cells per experiment) (Figure 7H). By contrast, in cells overexpressing cherry-KCC2, the increase of R_430/500_ in response to the application of KCl was 4.5 ± 0.5 units (Figures 7E–H). The application of Furosemide (1 mM) reduced KCC2-dependent change of R_430/500_ to 1.3 ± 0.1 units. Thus, these data introduce a novel way of recording KCC2 activity by estimating Cl^−^ influx. Our data also confirm the observations made in Figures 5D,E on the low activity of endogenous transporters in N2a cells, making them an attractive model to study exogenously expressed cation-Cl^−^ transporters.

Consistent with the results described in Figures 5, 6, the recording of the Cl-Sensor fluorescence at resting conditions revealed high variability of R_430/500_ ratio values ranging from 0.7 to 1.4 units in both mock transfected and cherry-KCC2 transfected cells (Figure 7F). Although the values obtained in individual cells largely overlapped in both experiments, there was a clear difference in mean values that was further confirmed using Kolmogorov–Smirnov test (*p* < 0.01, *n* = 3) (Figure 7F). Thus, despite the high level of variability, the quantitative analysis of R_430/500_ itself allows identifying cultures expressing active chloride extruder. Interestingly, the treatment of the cells expressing cherry-KCC2 with furosemide (1 mM) during 5 min (traces are not shown) did not affect significantly the R_430/500_ (Figure 7F). These results indicate that, at resting conditions, the [Cl^−^]_i_ is close to the equilibrium and inhibition of the potassium-chloride co-transporters does not produce rapid changes in [Cl^−^]_i_ and, consequently, in Cl-Sensor fluorescence.

### Calibration of the ratiometric recordings from cultured hippocampal neurons

**Figure 8:**
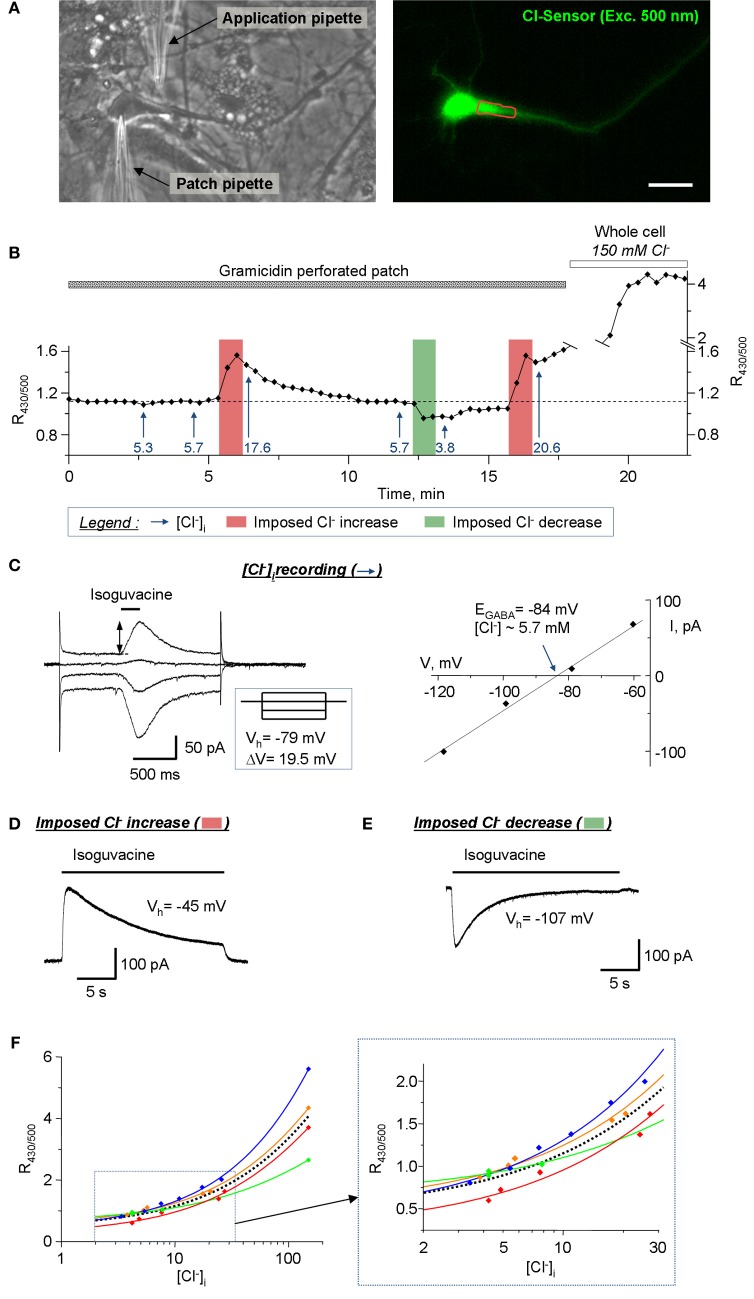
**Calibration of the Cl-Sensor ratiometric fluorescence using simultaneous imaging and gramicidin-perforated patch clamp recordings. (A)** Images of cultured hippocampal neuron taken with transparency light (left) and in fluorescence mode (right, excitation with 500 nm). The left image was taken after formation of the patch clamp gigaseal with patch pipette. The application pipette was placed as indicated. The right fluorescence image is one of images obtained during fluorescence recording illustrated in **(B)**. The red line shows ROI drawn for analysis of the fluorescence. Scale bar 20 μm. **(B)** Plot of R_430/500_ change during simultaneous imaging and patch clamp recording. The arrows show the estimated level of [Cl^−^]_i_, determined using E_GABA_ record protocol, at specified time of recording. The vertical bars indicate times of the imposed Cl^−^ increase or decrease as indicated in the legend. **(C)** Example of the recording of E_GABA_ reversal potential. The left plot shows neuron's responses to short applications of isoguvacine at different V_h_. The inset shows the scheme of V_h_ steps. E_GABA_ was determined as the point of crossing of voltage current relationship (VCR) of isoguvacine responses (right plot). The level of [Cl^−^]_i_ (5.3 mM in the illustrated example) was calculated using Nernst equation (E_GABA_ = −59.16 mV × log([Cl^−^]_o_/[Cl^−^]_i_), where E_GABA_ = −84 mV and [Cl^−^]_o_ = 150 Mm). **(D)** Procedure of the imposed Cl^−^ increase. The neuron was depolarised to −45 mV for 30s. During depolarization the 22 s jet of isoguvacine was applied through the application pipette inducing an outward ion current that carried Cl^−^ ions into the cell. **(E)** Procedure of the imposed Cl^−^ decrease. The neuron was hyperpolarized to −107 mV for 30 s. During depolarization the 22 s jet of isoguvacine was applied through the application pipette inducing an inward ion current and efflux of Cl^−^ from the cell. **(F)** Doze-dependences of R_430/500_. The colored lines show best sigmoid fitting curves for each individual experiment. The dotted black line shows the mean fitting curve. The inset shows the fragment of doze-dependence plot at higher resolution base. Notice the variability of dose-dependences in individual experiments.

Another important application for Cl-Sensor is the analysis of [Cl^−^]_i_ dynamics in neuronal cells. The estimated level of [Cl^−^]_i_ in mature pyramidal neurons is 4 mM (Tyzio et al., 2008), which is close to the limit of the sensitivity of Cl-Sensor (Waseem et al., 2010). To verify whether our conventional epifluorescence setup allows visualization of [Cl^−^]_i_ change in mature neurons, we performed a simultaneous recording of Cl-Sensor fluorescence and the reversal potential of GABA_A_ receptor-channel (GABA_A_R) mediated ion current (E_GABA_) (Figures 8A,B). To preserve the intracellular milieu, the E_GABA_ recordings were performed using gramicidin-perforated patch clamp as described previously (Chudotvorova et al., 2005; Pellegrino et al., 2011). All recordings were performed using voltage-clamp mode and holding membrane potential (V_h_ = −78 mV) that was close to the resting membrane potential (V_m_ ranged from −72 to −78 mV in different experiments). The estimation of E_GABA_ was performed at different moments of the experiment (mentioned with arrows on Figure 8B) by applying, through the application pipette, four consecutive 200 ms jets of external solution containing 30 μM isoguvacine, a selective agonist of GABA_A_R. Each pulse of isoguvacine was applied at different V_h_, which generated currents of different amplitudes and directions and allowed determining the value of E_GABA_ (Figure 8C). In our experimental conditions (HEPES-buffered saline, no bicarbonate ions added), the E_GABA_ corresponded to E_Cl_ that permitted direct calculation of [Cl^−^]_i_ using the Nernst equation. In the recorded neurons, the calculated [Cl^−^]_i_ values at resting conditions were ranging from 5.3 to 5.7 mM (Figure 8B). To induce the increase of [Cl^−^]_i_, a prolonged 20 s application of isoguvacine was applied to neurons at V_h_ = −45 mV. This generated an outwardly directed current (Figure 8D) and induced an increase of the R_430/500_ ratio of Cl-Sensor fluorescence (Figure 8B). The estimated levels of [Cl^−^]_i_ after first and second prolonged jets applications to depolarized neurons were 17.6 mM and 20.6 mM, respectively (Figure 8B). In opposite, the prolonged application of isoguvacine at −107 mV produced an inward ion current (Figure 8E) and induced clearly detectable decrease of R_430/500_ ratio. The calculated [Cl^−^]_i_ after this event was 3.8 mM (Figure 8B). The breakdown of the patch membrane at the end of the experiment (patch pipette contained 150 mM of KCl and 10 mM HEPES, pH 7.2) resulted in rapid and strong increase of R_430/500_ (Figure 8B) that not only allowed estimation of the working range of Cl-Sensor but also served as a control of the integrity of gramicidin perforated patch during the experiment.

Thus, the ratiometric measurement of Cl-Sensor fluorescence, using conventional epifluorescence microscope, allows visualization of R_430/500_ changes in response to activation of GABA_A_R at low concentrations of [Cl^−^]_i_, close to physiological conditions. Similar changes of R_430/500_ were recorded in three other experiments (Figure 8F). It should be noticed that, while in all four experiments we observed similar negative and positive shifts of the R_430/500_ in response to imposed decrease or increase of [Cl^−^]_i_, the absolute values of R_430/500_ at similar levels of [Cl^−^]_i_ varied from experiment to experiment (Figure 8F). Particularly, the variability of R_430/500_ was more pronounced at high levels of [Cl^−^]_i_ (20–150 mM). This observation is consistent with the variability of R_430/500_ and Δ R/R described above (Figures 5–7) and should be taken into account during design of the experiments.

### Stable recording of Cl-Sensor fluorescence from neuronal dendrites and spines

**Figure 9:**
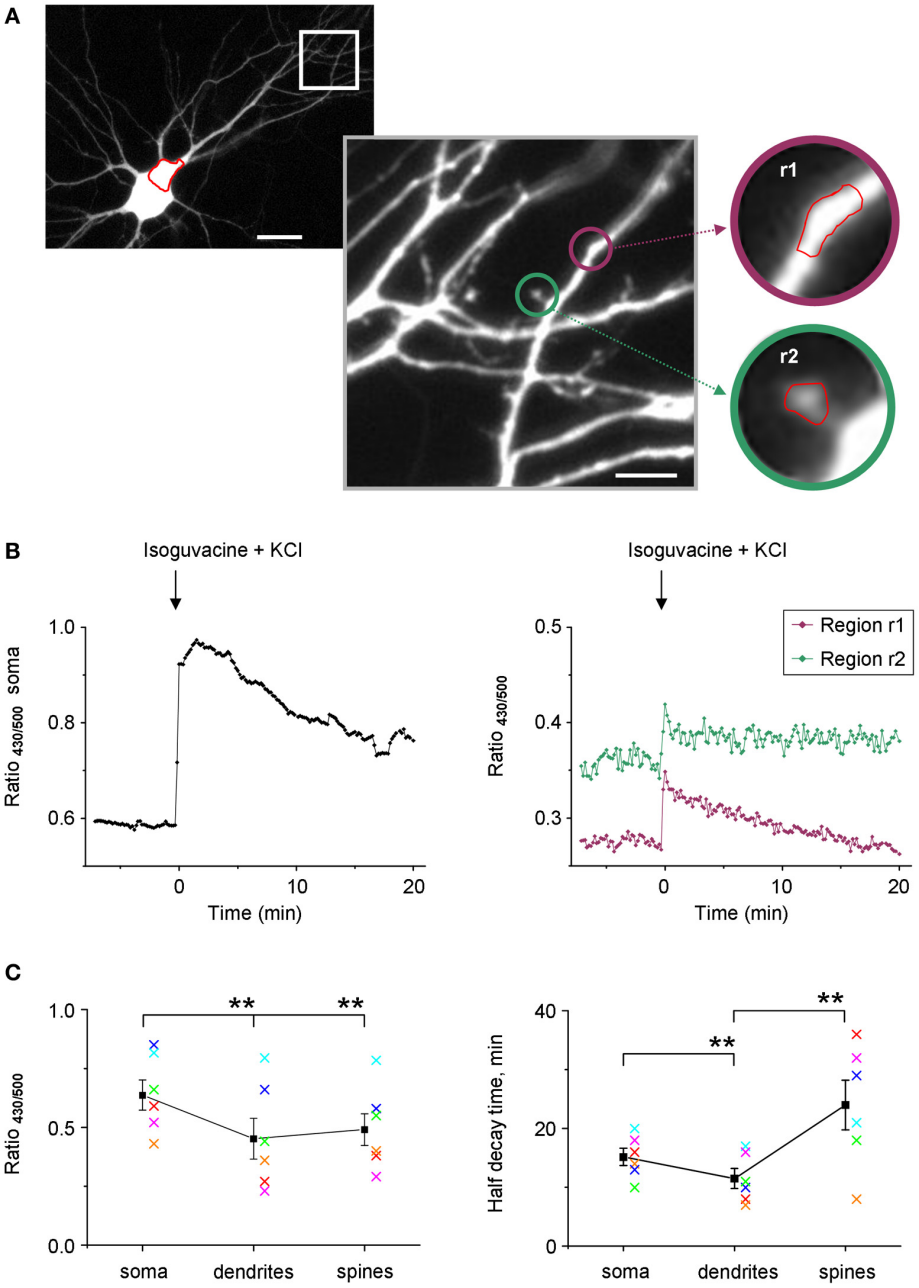
**Stable recording of Cl-Sensor fluorescence from neuronal dendrites and spines. (A)** Cultured hippocampal neuron expressing Cl-Sensor (13 days *in vitro*, E19 embryos). The selected dendrite region is shown in greater digital magnification. This image represents one of original sequential images taken during fluorescence measurements (500 nm excitation filter). Scale 20 μm in left panel and 5 μm in right panel. The insets illustrate regions of interest (r1 and r2) drawn on a dendrite and on a spine for analysis of fluorescence dynamics. **(B)** Examples of ratio R_430/500_ recordings from soma (left plot) and two different regions (right plot) indicated in panels (A). The increase of [Cl^−^]_i_ was induced by a 25 s application of an external solution containing 25 mM KCl and 30 μM isoguvacine, an agonist of GABA_A_ receptors. Arrow indicates moment of application. **(C)** Mean ± SEM of R_430/500_ (left plot) and half decay times obtained from experiments similar to one illustrated in (**A** and **B**). ^**^p < 0.01. The values obtained in each experiment are coded with the same color in left and right plots.

Taking into account the weak use-dependency of F_500_ fluorescence of Cl-Sensor (Figure 3A), we increased the time of YFP_Cl_ exposure in the next set of experiments on cultured hippocampal neurons that allowed visualization of tiny dendrites and spines (Figure 9A). In the illustrated example, the R_430/500_ was successfully recorded using an exposure time of 200 ms for F_500_ (image binning 2 × 2) and 50 ms for F_430_ (image binning 8 × 8). Under such recording conditions, the basal line of R_430/500_ remained stable for 8 min and was higher in soma than in dendrites and spines (Figure 9B). The difference of R_430/500_ between soma and dendrites was detected in five other experiments made on neurons from different culture preparations suggesting higher [Cl^−^]_i_ in soma (Figure 9C). The brief 25 s application of an external solution, containing 25 mM KCl and 30 μM isoguvacine, induced a strong and rapid increase of R_430/500_ in neuronal soma, dendrites, and spines, reflecting presumably increase of [Cl^−^]_i_. After this increase, the level of R_430/500_ started to recover slowly in all neuronal compartments. The half times of the recovery were significantly shorter in tiny dendrites than in soma and were significantly longer in spines than in dendrites (Figure 9C).

## Discussion

The main finding of this work is that, following a 430 nm excitation, there is a strong inactivation of the fluorescence emitted by the YFP_Cl_ component of Cl-Sensor. We also found that the increased sensitivity of Cl-Sensor to 430 nm light is due to mutations rendering YFP more sensitive to Cl^−^. Although the exact mechanism of inactivation is unknown, this finding let us making the adequate adjustments to the existing setup and obtaining reliable measurements of the Cl-Sensor fluorescence ratio. A future detailed study is necessary to elucidate the mechanisms of YFP_Cl_ inactivation by 430 nm light.

The Cl-Sensor is a chimera protein composed of two components, Cl^−^ insensitive CFP and Cl^−^ sensitive YFP_Cl_ connected with a polypeptide linker of 20 amino acids. The enhanced Cl^−^ sensitivity of YFP is due to the inclusion of three mutations, H148Q, I152L, and V163S (Markova et al., 2008), chosen based on previous reports by Galietta et al. (2001) and Jayaraman et al. (2000). A detailed spectroscopy analysis of mutants H148Q (Jayaraman et al., 2000) and I152L (Galietta et al., 2001) revealed that the Cl^−^ sensitive absorbance region is located within 460 to 540 nm wavelengths of the visible light spectrum (peak at 513 nm). Accordingly, the present work was performed using a recently commercialized filter set that permits the excitation of YFP close to the peak of its maximal chloride sensitivity (490–510 nm). We found that Cl-Sensor excited at 500 nm shows low rate of use-dependent inactivation, allows stable recording of fluorescence at resting conditions and shows strong decrease in fluorescence (from 10 to 60% in individual cells) during an imposed rise of [Cl^−^]_i_. Thus, changes in [Cl^−^]_i_ can be effectively visualized using excitation with a 500 nm filter. Excitation at 430 nm of the reference CFP component offers the advantage of recording of the YFP signal normalized to the level of protein expression and, thus, allows a quantitative comparison of the Cl^−^ dependent changes in different cells and experiments. The filter set used here allowed cumulating the fluorescence from both the CFP and YFP components of Cl-Sensor. In this configuration F_430_ included both the Cl^−^ resistant component emitted by CFP and the Cl^−^ sensitive component emitted by YFP_Cl_ (see Figure 1 for spectra and Figure 4 for F_430_ responses). A more advanced configuration of the setup would consist in recording separately the CFP (excitation at 430 nm and emission at 480 nm) and YFP_Cl_ signals (excitation at 516 nm and emission at 535 nm).

The existence of a use-dependent photoinactivation of Cl-Sensor was known in previous works. Thus, Markova et al. (2008), Waseem et al. (2010), and Bertollini et al. (2012) used short excitation times, a low intensity excitation source and high sensitivity CCD camera. In the present work, we recorded the YFP fluorescence using relatively long excitation with 500 nm light and applied short 430 nm pulses to obtain the reference images at high binning (low spatial resolution).

Regardless of the exact mechanisms of the photoinactivation of Cl-Sensor, our work emphasized the importance of the selection of optimal recording parameters for each fluorescence setup. The application of the recording paradigm illustrated in Figure 3 would allow easy and rapid determining of the origin of photoinactivation and settling the optimal recording parameters.

### Cl-Sensor ratiometric fluorescence and intracellular Cl^−^ concentration

We show here the effectiveness of the proposed modifications of the lightpass and recording protocol for stable long-lasting ratiometric recording of Cl-Sensor emitted fluorescence using conventional epifluorescence setup. As examples, we characterize Cl-Sensor ratiometric responses to activation of chloride-permeable ion channels and/or potassium-chloride co-transporters in heterologous expression system and cultured hippocampal neurons. Moreover, we provide an experimental protocol allowing long-lasting imaging of the kinetic of Cl-Sensor fluorescence in subcellular neuronal structures such as tiny dendrites and spines. These two subjects are discussed in details below. The third important application for Cl-Sensor is the estimation of [Cl^−^]_i_. Using simultaneous patch clamp-recording and Cl-Sensor imaging, we demonstrated that Cl-Sensor ratiometric imaging allows the visualization of as little as 2 mM changes of [Cl^−^]_i_ from its resting level of 5–7 mM (Figure 8). By refilling the recorded neurons using a solution containing 150 mM KCl, we determined also a large dynamic range of the Cl-Sensor from 0.7 relative units at 5 mM [Cl^−^]_i_ to 4.0 units at 150 mM [Cl^−^]_i_ (data from mean doze-dependence curve in Figure 8F). Thus, using determined dose-dependence curve, one can easily make a crude estimation of [Cl^−^]_i_.

During simultaneous imaging and patch-clamp recordings, we observed a high variability of the absolute values of R_430/500_ corresponding to nominally similar concentrations of Cl^−^. Similar variability in the resting R_430/500_ was found in cultured hippocampal neurons that were not used for the patch clamp recording (Figure 9C) and in cultured N2a cells (Figures 5C,D). These data are consistent with previous observations also showing high variability of the ratiometric measurements of Cl^−^ sensitive proteins in dose-dependence experiments (Kuner and Augustine, 2000; Waseem et al., 2010). The mechanisms underlying such high variability of ratiometric responses are not known. The YFP protein, in addition to its high Cl^−^ sensitivity, is also sensitive to some intracellular energy metabolites (acetate and formate) and pH (Jayaraman et al., 2000). In consequence, the modifications in pH_i_ and/or energy metabolism in different cells might contribute to changes in the YFP fluorescence and influence the basal level of R_430/500_. In keeping with this, the measurement of the exact level of [Cl^−^]_i_ using Cl-Sensor requires development of novel calibration protocols that will take into account a large number of variables, including pH, energy metabolism, ionic strength, and other important parameters affecting Cl-Sensor fluorescence.

### N2a cells expressing Cl-Sensor: a model to study the ion-transport ability of the cation-chloride co-transporter KCC2

One of the most important applications for Cl-Sensor is the estimation of the Cl^−^ transport activity of KCC2 and its mutants. KCC2 is a neuron specific potassium-chloride co-transporter maintaining a low level of [Cl^−^]_i_ in mature neurons. The activity of this transporter changes under several pathological conditions leading to the modification of [Cl^−^]_i_ and Cl^−^ dependent change of the inhibitory strength of GABA and glycine (Medina and Chudotvorova, 2006; Kahle et al., 2008; Blaesse et al., 2009). Consequently, it is of primary importance to understand the signaling pathways controlling KCC2 and find molecules capable of compensating for changes in KCC2 activity during pathology. As KCC2 is an electroneutral transporter carrying K^+^ and Cl^−^ ions in stoichiometry 1:1 (Payne, 1997), one of the most efficient ways of measuring KCC2 activity consists in determining its contribution to the resting level of [Cl^−^]_i_ as well as to the Cl^−^ extrusion capacity of the cell (Chudotvorova et al., 2005; Lee et al., 2005; Akerman and Cline, 2006; Inoue et al., 2006). In the cited studies (as well as in many later works), the analysis of resting [Cl^−^]_i_ and kinetics of KCC2-dependent Cl^−^ changes were performed using gramicidin-perforated patch-clamp recordings. In addition to the patch-clamp approach, we have recently reported a successful attempt at visualizing KCC2-dependent changes in [Cl^−^]_i_ using non-invasive ratiometric measurements of Cl-Sensor emitted fluorescence (Pellegrino et al., 2011).

In the present study, we describe three complementary ways of estimating the activity of KCC2 using Cl-Sensor as probe (Figure 5):

1. The first approach is the analysis of the resting level of R_430/500_. N2a cells expressing exogenous KCC2 show significantly lower levels of R_430/500_ corresponding to lower levels of [Cl^−^]_i_; this is consistent with previous reports performed on different cell lines and cultured neurons (Chudotvorova et al., 2005; Lee et al., 2005; Akerman and Cline, 2006; Inoue et al., 2006; Pellegrino et al., 2011). Although we observed a highly reproducible and statistically significant difference between control (mock) and KCC2 transfected cells in 100% of experiments performed on different cell lines (*n* > 50, I.M., P.F.), the analysis of resting [Cl^−^]_i_ remains an indicative approach as this parameter depends on the equilibrium between many contributing components.
2. The second approach is the analysis of the kinetics of Cl^−^ extrusion after artificially induced Cl^−^ increase through GlyR. We found that N2a cells are particularly appropriate for this test as they do not express detectable functional endogenous furosemide-sensitive CCCs. Once increased, [Cl^−^]_i_ remains elevated in these cells for tens of minutes; by contrast, cells overexpressing KCC2 rapidly extrude Cl^−^. This rapid extrusion is KCC2 specific as it is almost fully inhibited with 1 mM furosemide. The advantage of the “Cl^−^ extrusion approach” is that it allows an estimation of the Cl^−^ extrusion activity of KCC2 at the moment of study and, thus, it is close to physiological conditions when in experimental conditions. It also allows comparative pharmacological tests on the same cells (application of furosemide in present study). In addition, all cells (mock and expressing KCC2) extrude Cl^−^ from nominally similar increased levels achieved by activation of GlyR channels under depolarized conditions. The main inconvenience of this approach is the relatively long time necessary to carry out a full experiment (from 30 min to few hours) and the possible activation of Cl^−^ dependent signaling pathways known to modify the activity of KCC2.
3. The third approach consists in measuring Cl^−^ influx through KCC2 operating in the reverse direction (Cl^−^ influx test). As KCC2 operates in the direction of the summed chemical gradient of K^+^ and Cl^−^, an increase of the extracellular K^+^ ([K^+^]_o_) leads to the reversal of KCC2 and the pumping of Cl^−^ into the cell (Payne, 1997). In agreement with this, we found that in cells expressing KCC2, the simple increase of [K^+^]_o_ (in absence of the activation of GlyR) leads to a strong increase of R_430/500_ that is fully blocked by furosemide. Interestingly, in cells that do not express KCC2, changes in R_430/500_ are almost negligible, further confirming the low level of the activity of endogenous KCCs. The advantage of the Cl^−^ influx test is that it allows a rapid estimation (within a dozen minutes) of the net activity of KCC2. The disadvantage is that KCC2 operates in reverse direction and, thus, far from physiological conditions. Moreover, the strength of Cl^−^ influx depends on at least two parameters: expression level of KCC2 and Cl^−^ gradient. Under other equal conditions, Cl^−^ influx will be stronger in cells with low [Cl^−^]_i_. To circumvent this problem, we applied the maximum possible concentration of KCl (140 mM) outside of cells, creating constant intracellularly directed gradients for both K^+^ and Cl^−^ (assuming that [K^+^]_i_ is in range of 100–150 mM and [Cl^−^]_i_ is in range of 7–50 mM). A further specific analysis is required to make a comparison between the efficacy of the estimation of KCC2 activity using “Cl^−^ extrusion” and “Cl^−^ influx” tests.

### Cl-Sensor fluorescence in tiny neuronal structures

The analysis of the [Cl^−^]_i_ kinetics in different neuronal structures, including axons, dendrites, and spines, is one of the priorities in the field of neurobiology (Khirug et al., 2008; Pellegrino et al., 2011). Our finding of the low degree of photoinactivation of Cl-Sensor during excitation at 500 nm permitted high-resolution images of neurons and an analysis of the dynamics of R_430/500_ in soma, dendrites, and even dendritic spines. We found that the kinetics of R_430/500_ recovery differ in tiny dendrites and spines, opening new perspectives for the study of chloride homeostasis in these neuronal structures.

### The optimized protocol and recommendations for reliable Cl^−^ monitoring using Cl-Sensor

1. Avoid cell illumination with 430 nm light prior to beginning of the experiment. This is the main recommendation to preserve the stability of Cl-Sensor fluorescence.
2. Make pre-acquisition adjustments using transparency mode. The selection of the field of interest and focusing should be performed using transparency mode with low intensity visible light and employing cut off filter (<480 nm) introduced into the light pass. The final adjustment of the focus could be made with excitation of the Cl-Sensor at 500 nm using single image acquisition but never “life imaging mode” as to avoid bleaching of the YFP.
3. Select the optimal acquisition parameters at 500 nm excitation in order to obtain high-resolution images with no bleaching of the signal (protocol illustrated in Figure 3A).
4. Select the optimal acquisition parameters at 430 nm excitation. Perform time-lapse recording using only 500 nm excitation and introduce, during the recording, a single 430 nm excitation event in order to select the longest time of excitation that does not affect 500 nm evoked fluorescence (protocol illustrated in Figure 3C). For *advanced users:* the most reproducible results could be obtained when ratio R_430/500_ is close to 1. Given that the intensity of 430 nm induced fluorescence is limited by its phototoxicity, we used higher binning when taking images at 430 nm excitation. The MDA option of the Metamorph software allows the easy alignment and arithmetic operations with images taken with different pixels binning.
5. For ratiometric recordings, first excite Cl-Sensor at 500 nm immediately followed by 430 nm. Do not alternate the order of excitation (see protocol illustrated in Figure 4).

In summary, we characterized the novel process of photoinactivation of the Cl-Sensor by illumination at short blue spectrum light. Based on this observation, we made modifications to the conventional epifluorescence setup in order to obtain long-lasting stable ratiometric imaging of Cl^−^ dynamics in cell lines and cultured neurons. We describe the proposed modifications and provide detailed protocol for ratiometric measurement of Cl-Sensor fluorescence.

Thus, the ratiometric measurement using Cl-Sensor is a good reliable approach for measurement of the dynamic of Cl-changes in range from 2 to 150 mM and crude estimation of [Cl^−^]_i_. The estimation of the exact level of [Cl^−^]_i_ requires the development of novel protocols taking into account large number of parameters and calibration of each recorded cell.

### Conflict of interest statement

The authors declare that the research was conducted in the absence of any commercial or financial relationships that could be construed as a potential conflict of interest.
